# Clinical features and management of posttraumatic subperiosteal hematoma of the orbit

**DOI:** 10.4103/0301-4738.73721

**Published:** 2011

**Authors:** Usha R Kim, Vipul Arora, Akash D Shah, Urvashi Solanki

**Affiliations:** Aravind Eye Hospital, Madurai, Tamil Nadu, India

**Keywords:** Steroids, subperiosteal hematoma, traumatic

## Abstract

Traumatic subperiosteal hematoma (SpH) usually presents late, after the initial trauma. It is generally seen in young males. Computed tomography is the best mode of imaging and helps to rule out orbital fracture or associated subdural hematoma. We present the clinical features and management of four patients seen at the orbit clinic with SpH. Management is based on time of presentation, visual acuity and any communicating bleed. The prognosis of traumatic SpH is excellent if treated with an individualized patient approach.

Subperiosteal hematomas (SpH) of the orbit typically are a result of blunt trauma to the head region, although there are case reports of spontaneous and congestive hematoma. They are the orbital equivalent of an intracranial epidural hematoma. Congestive hemorrhages are induced by increased intra-abdominal and intrathoracic pressure as in Valsalva maneuver, labour or scuba diving.[1] Spontaneous hemorrhages can occur in association with blood dyscrasias, including hemophilia, scurvy, leukemia[1] and sinusitis.[2] In this case series, we present the clinical features and management of our patients presented with traumatic SpH and recommend a management protocol.

## Case Reports

### Cases 1 and 2

**Figure 1:**
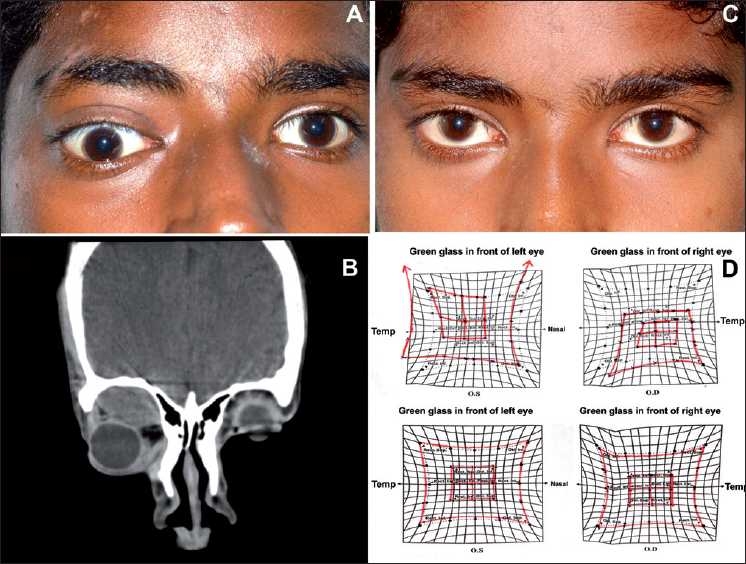
(A) Clinical photograph of the patient showing proptosis and downward displacement of the globe. (B) Computerized tomography scan photograph (coronal) showing a hypodense lesion measuring 3 × 2 × 1.2 cm superior to the optic nerve and superior rectus muscle. (C) Clinical photograph of the same patient 1 month after treatment of steroids showing complete resolution of proptosis. (D) Hess charting upper image showing restriction of movements in the right eye at the time of presentation and lower image showing normal movements after treatment

Two similar cases of a 16-year-old and a 14-years-old male presented with protrusion of the right and left eye, respectively, with vertical diplopia, after sustaining trauma following a fall from height 20 and 10 days prior, respectively [Fig. 1A]. Best corrected visual acuity (BCVA) of both eyes of both the patients was 20/20. On examination, the affected eye had eccentric proptosis with eye down and out. Extra ocular movements (EOM) were restricted in elevation and adduction. Hess charting was performed, which confirmed the motility defect. A neurological examination revealed no abnormality. A computed tomography (CT) scan showed a hypodense lesion located superior to the optic nerve and superior rectus muscle and posteromedial to the globe with no bony discontinuity or fracture [Fig. 1B] thus confirming the diagnosis of posttraumatic SpH in both patients. Patient one was taken for aspiration of the hematoma, but it resulted in dry aspiration. The patient was started on intravenous cefotaxime (50 mg/kg body weight in divided doses 12 hourly) and intravenous dexamethasone (4 mg twice a day) for 5 days. Patient two was directly started on intravenous cefotaxime (50 mg/kg body weight in divided doses 12 hourly) and intravenous dexamethasone (4 mg twice a day).

There was reduction of symptoms after five days and both the patients were shifted on oral steroids (tablet prednisolone 1 mg/kg body weight in divided doses). After 1 month there was complete resolution of proptosis with the Hess chart showing normal EOM [Figs. 1C and D].

### Case 3

**Figure 2:**
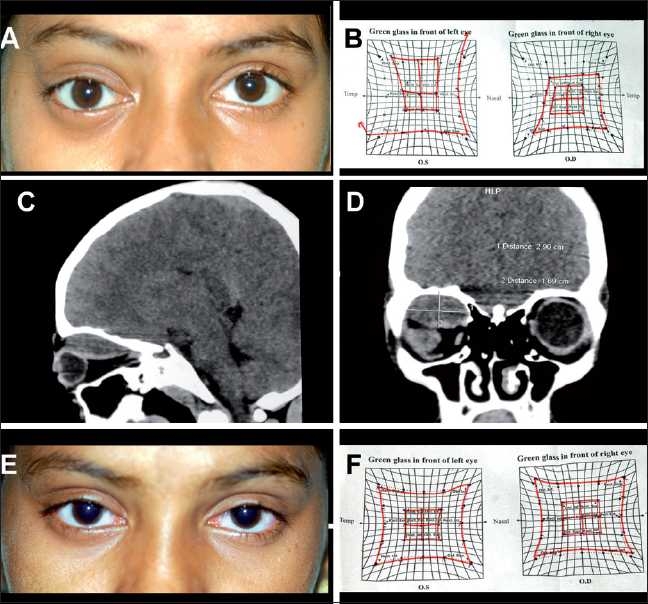
(A) Clinical photograph of the patient showing proptosis and downward displacement of the globe. (B) Hess charting showing restriction of movements in the right eye. (C and D) Computerized tomography scan photograph (coronal and sagittal) showing a hypodense lesion in the superior quadrant of the right orbit measuring 3.2 × 2.7 × 1.4 cm, pushing the globe down and out. (E) Photograph of the same patient 1 week after aspiration of the hematoma. (F) Hess charting showing normal movements after treatment

A 12-year-old female presented with dimness of vision and protrusion of the right eye since the past 3 days following a fall from her bicycle. BCVA of the right eye was 20/200 and of the left eye was 20/20. On examination, the patient had right eccentric proptosis with supra- and infra-orbital fullness [Fig. 2A]. EOM showed restriction on elevation and adduction. Hess charting confirmed the motility defects [Fig. 2B]. CT scan showed a hypodense lesion in the superior quadrant of the right orbit pushing the globe down and out [Fig. 2C and D]. A diagnosis of posttraumatic SpH was made. The patient underwent aspiration of the SpH. Under intravenous sedation (injection midazolam 0.05 mg/kg body weight), an 18-gauge, 1.5-inch needle was passed transcutaneously lateral to the supraorbital notch till blood was seen at the hub of the needle. About 5 cc of dark red blood was aspirated, which resulted in immediate reduction of proptosis. On the first postoperative day, the visual acuity was 20/20 with no proptosis and normal extraocular movements [Figs. 2E and F].

### Case 4

A 15-year-old boy presented with left-sided proptosis, pain and diplopia for the past 6 days. Thirteen days prior he sustained head injury after a fall from a tree. After 6 days, he developed gradually progressive left eye proptosis, pain and diplopia.

**Figure 3:**
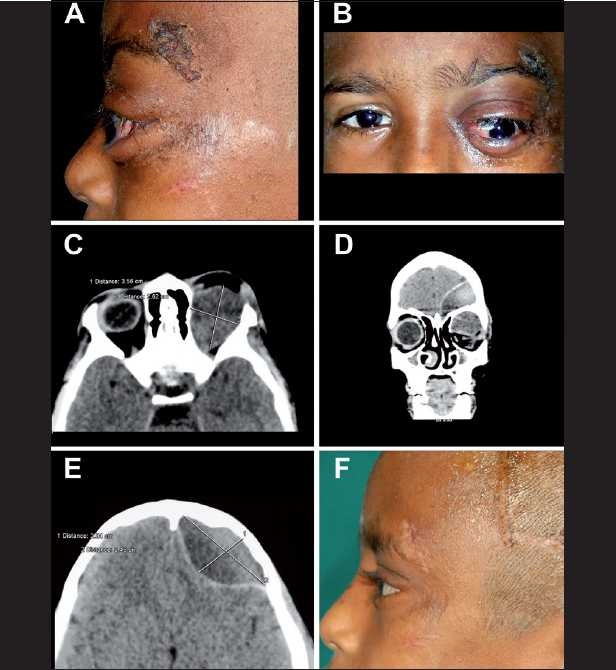
(A and B) Clinical photograph of the patient showing the nonaxial proptosis and exposure keratopathy in the left eye with a lacerated wound over the left supraorbital ridge. (C, D and E) Computerized tomography scan (axial and coronal) showing superomedial orbital subperiosteal hematoma measuring 3.44 × 2.73 × 2.24 cm, in continuity with the subfrontal extradural hematoma measuring 5.75 × 4.53 × 3.30 cm through the undisplaced orbital roof fracture in the left side and displacing the globe downwards, laterally and forward. (F) Postoperative photograph 1 week after frontal craniotomy and superior orbitotomy showing complete resolution of proptosis

The visual acuity in the right eye was 20/40 and in the left was 20/200. On the left, there was an eccentric proptosis of 6 mm [Figs. 3A and B]. There was exposure keratopathy. Pupils were normal and reacting. There was marked limitation of elevation and depression, slight limitation of abduction and adduction. CT scan demonstrated a superomedial orbital SpH [Figs. 3C and D] in continuity with subfrontal extradural hematoma through the undisplaced orbital roof fracture in the left side [Figs. 3D and E].

The patient underwent left frontal craniotomy with superior orbitotomy with evacuation of the blood clot from both the sites. The patient made an uneventful recovery with no neurological deficit, normal vision and fundus [Fig. 3F].

## Discussion

Traumatic SpH of the orbit commonly occurs in young patients.[2] It almost always presents in the superior orbit and usually presents late where there is gross hypoglobus, dimness of vision, intractable diplopia or pain. These hematomas develop between the bone and separated periosteum in the orbital roof. This usually occurs as a result of direct rupture of the subperiosteal blood vessels or as an extension of a subgaleal hematoma.[3]

All our patients had trauma in the head region. The first two patients presented late while the third patient presented early, within 3 days after the initial trauma. The fourth patient presented with delayed orbital hemorrhage after 7 days of initial trauma. The timing of delayed hemorrhage may be related to the fibrinolysis and clot retraction that occur during this period.[4]

CT is the best technique as it delineates the size and extent of the hematoma and demonstrates any associated orbital wall fractures. Signs on a CT scan include (a) sharply defined, high-attenuation mass (blood density) with a broad base abutting the superior orbital roof, (b) inferior displacement of the orbital contents and (c) optic nerve stretching.[5] The differential diagnosis includes neoplasms and inflammation. However, when the clinical presentation is combined with the CT, a diagnosis should be easily established. The other imaging modalities that help in diagnosis are magnetic resonance imaging (MRI) and angiography. MRI of SpH reveals a biconvex, well-defined mass of varied signal intensity, depending on the age of the hematoma. T1-weighted images will show a low signal in the hyperacute stage (fresh blood) and a high signal in the subacute stage (3–7 days, before cell lysis). T2-weighted images will show a high signal in the hyperacute stage and a low signal in the subacute stage.[6] Angiography can demonstrate stretching and inferior displacement of the ophthalmic artery on the affected side. The absence of orbital venous anomalies helps separate patients with SpH from those with spontaneous hemorrhage. Angiography may also be used in the diagnosis of a carotid cavernous fistula.[5]

Conservative management is generally recommended, but severe visual disturbance requires surgical intervention. Management options include observation, needle aspiration and surgical evacuation.[7] Patients presenting early and having associated ocular problems can be immediately taken for aspiration as they have liquid blood. This management was performed in case 3, wherein the patient presented early, where aspiration was carried out that resulted in complete resolution of the hematoma with no recurrence. Patients presenting late generally have clotted blood, which causes dry aspiration, as seen in case 1. Such patients with no ocular compromise can be started on intravenous steroids as in our first two cases, which resulted in resolution of all the symptoms within 6 weeks.

It is possible that the steroid pulse therapy was dramatically effective in early resolution of the hematoma, as in our case 3. The mechanism is due to the reduction of edema and the suppression of cytotoxic humoral factors such as free radicals and cytokines.[8] However, patients who present late with visual compromise or patients with associated fracture roof of orbit or subgaleal hematoma can be directly taken for surgical drainage. Drainage has been performed successfully through needle aspiration and surgical evacuation. Needle aspiration is less invasive, but does not remove clots or stop active bleeding.[7,9] Orbital exploration allows removal of coagulated blood, drain placement and fracture repair.[7]

Thus, to conclude, SpH of the orbit must be considered in the differential diagnosis of unilateral proptosis after trauma. The radiographic features, in the proper clinical setting, can lead to early diagnosis and prevent late sequelae. Steroids form an important tool in early resolution of hematoma, specially when there is a delayed presentation.
